# Modulation of the Bi^3+^ 6s^2^ Lone Pair State in Perovskites for High‐Mobility p‐Type Oxide Semiconductors

**DOI:** 10.1002/advs.202104141

**Published:** 2022-01-07

**Authors:** Jueli Shi, Ethan A. Rubinstein, Weiwei Li, Jiaye Zhang, Ye Yang, Tien‐Lin Lee, Changdong Qin, Pengfei Yan, Judith L. MacManus‐Driscoll, David O. Scanlon, Kelvin H.L. Zhang

**Affiliations:** ^1^ State Key Laboratory of Physical Chemistry of Solid Surfaces Collaborative Innovation Center of Chemistry for Energy Materials College of Chemistry and Chemical Engineering Xiamen University Xiamen 361005 China; ^2^ Department of Chemistry and Thomas Young Centre University College London London WC1H 0AJ UK; ^3^ MIIT Key Laboratory of Aerospace Information Materials and Physics College of Science Nanjing University of Aeronautics and Astronautics Nanjing 211106 China; ^4^ Department of Materials Science and Metallurgy University of Cambridge 27 Charles Babbage Road Cambridge CB3 0FS UK; ^5^ Diamond Light Source Ltd. Harwell Science and Innovation Campus Didcot OX11 0DE UK; ^6^ Beijing Key Laboratory of Microstructure and Property of Solids Faculty of Materials and Manufacturing Beijing University of Technology Beijing 100124 China

**Keywords:** DFT calculations, electronic structures, p‐type oxide semiconductors, photoemission spectroscopy

## Abstract

Oxide semiconductors are key materials in many technologies from flat‐panel displays，solar cells to transparent electronics. However, many potential applications are hindered by the lack of high mobility p‐type oxide semiconductors due to the localized O‐2p derived valence band (VB) structure. In this work, the VB structure modulation is reported for perovskite Ba_2_Bi*M*O_6_ (*M* = Bi, Nb, Ta) via the Bi 6s^2^ lone pair state to achieve p‐type oxide semiconductors with high hole mobility up to 21 cm^2^ V^−1^ s^−1^, and optical bandgaps widely varying from 1.5 to 3.2 eV. Pulsed laser deposition is used to grow high quality epitaxial thin films. Synergistic combination of hard x‐ray photoemission, x‐ray absorption spectroscopies, and density functional theory calculations are used to gain insight into the electronic structure of Ba_2_Bi*M*O_6_. The high mobility is attributed to the highly dispersive VB edges contributed from the strong coupling of Bi 6s with O 2p at the top of VB that lead to low hole effective masses (0.4–0.7 *m*_e_). Large variation in bandgaps results from the change in the energy positions of unoccupied Bi 6s orbital or Nb/Ta d orbitals that form the bottom of conduction band. P–N junction diode constructed with p‐type Ba_2_BiTaO_6_ and n‐type Nb doped SrTiO_3_ exhibits high rectifying ratio of 1.3 × 10^4^ at ±3 V, showing great potential in fabricating high‐quality devices. This work provides deep insight into the electronic structure of Bi^3+^ based perovskites and guides the development of new p‐type oxide semiconductors.

## Graphical abstract

The development of high mobility p‐type oxide is crucial for many technologies including flat‐panel displays, solar cells, and transparent electronics. In this work, the development is reported of novel p‐type oxide semiconductors Ba_2_Bi*M*O_6_ (M = Bi, Nb, Ta) with high hole mobility up to 21 cm^2^ V^−1^ s^−1^, and variable bandgaps from 1.5 to 3.2 eV by modulating their VB structure via the Bi 6s^2^ lone pair states.

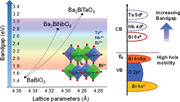

## 1 Introduction

Oxide semiconductors are being extensively used as key materials in modern optoelectronics technologies including flat panel displays, solar cells, light emitting diodes, and transparent electronics.^[^ ^1^ ^]^ Oxides such as ZnO, In_2_O_3_, Ga_2_O_3_, and SnO_2_ are typical wide bandgap n‐type semiconductors, in which the filled oxygen 2p^6^ orbitals form the valence band (VB), and the spatially extended metal s orbitals form the conduction band (CB), giving rise to a dispersive CB with a high electron mobility. In contrast, the development of high mobility p‐type oxide semiconductors remains challenging.^[^ ^2^ ^]^ The lack of p‐type oxide semiconductors severely hinders the development of many crucial technologies, e.g., p‐channel thin film transistors (TFT) and CMOS inverters for high‐resolution energy‐saving displays; p‐type semiconductor layers for efficient hole transport in photovoltaics and visible‐light active photocathodes for solar water splitting.^[^ ^2^, ^3^ ^]^

The difficulty in realizing high mobility p‐type oxide semiconductors arises from the electronic structure of oxides: the localized O 2p‐derived VB maximum (VBM) leading to difficulty in introducing shallow acceptors and large hole effective masses.^[^ ^2^, ^4^ ^]^ A strategy to mitigate the low mobility problem is to use the hybridization of O 2p with partially occupied d orbitals of transition metals (TM) to modulate band dispersion at the top of VB.^[^ ^5^ ^]^ TM oxides with closed‐shell Cu 3d^10^ (Cu*M*O_2_, *M* = Al, Cr, etc.), quasi‐closed shell d^6^ (Zn*M*_2_O_4_, *M* = Co, Rh, Ir) and d^3^ configurations (LaCrO_3_) have been explored as p‐type semiconductors.^[^ ^5^, ^6^ ^]^ However, these oxides still show limited improvement in the hole mobility (<5 cm^2^ V^−1^ s^−1^) or instability issue.^[^ ^7^ ^]^ An alternative approach is to use the ns^2^ lone pair orbitals from heavy post‐transition metal cations (e.g., Sn^2+^, Bi^3+^, Pb^2+^) to interact with O 2p, forming the filled antibonding states at the top of VB. The spatially extended *s* orbitals work more effectively to delocalize the hole states at the VB edge than TM d orbitals. This leads to more dispersive VBM and much smaller hole effective masses for high p‐type mobility. Following this concept, SnO is one of the most promising p‐type oxides owing to the dispersive Sn 5s O 2p derived VB. High hole mobility up to 30 cm^2^ V^−1^ s^−1^ have been reported for SnO bulk polycrystals and ≈10 cm^2^ V^−1^ s^−1^ for epitaxial thin films.^[^ ^8^ ^]^ SnO‐based p‐type TFTs and CMOS inverters were also investigated. However, phase instability of SnO still presents significant technical challenges for thin‐film synthesis and device integration.^[^ ^8^, ^9^ ^]^ The presence of Pb 6s^2^ lone pair electrons in halide perovskites is also the key factor for their appealing optoelectronic properties as photovoltaic materials.^[^ ^10^ ^]^

Bi^3+^ based oxides are also receiving increasing attentions as p‐type oxide semiconductors. Many Bi^3+^ based oxides show p‐type conductivity owing to the native Bi vacancies or aliovalent doping.^[^ ^11^ ^]^ The involvement of Bi^3+^ 6s^2^ lone pair states at VB could also provide a dispersive VBM for hole transportation, leading to high p‐type mobility, as demonstrated in the cases of BiVO_4_ and p‐type BiOI.^[^ ^12^ ^]^ To unleash the potential of *s*‐orbital chemistry of Bi^3+^ for p‐type oxide semiconductors, we turn to Bi‐based perovskites. The perovskites have the advantage of structural and compositional flexibility to allow design of novel multifunctional materials via doping or alloying. BaBiO_3_ has previously received much attention, because of its superconductivity upon doping with Pb or K.^[^ ^13^ ^]^ BaBiO_3_ actually has the structure of Ba_2_Bi^3+^Bi^5+^O_6_, due to the charge disproportionation of 2Bi^4+^ → Bi^3+^ + Bi^5+^. The Bi 6s^2^ lone pair state arising from Bi^3+^ could hybridize with O 2p to form the top of VB, of potential to provide a high p‐type mobility, whereas the empty Bi 6s^0^ from Bi^5+^ form the bottom of CB. Although no consensus has been reached on the exact values of its bandgap (varying from 0.3 to 2.0 eV),^[^ ^14^ ^]^ several previous works reported that BaBiO_3_ is a small bandgap semiconductor. On the other hand, as a result of the structural flexibility of perovskite, the Bi^5+^ could be replaced with other pentavalent cations such as Nb^5+^ or Ta^5+^ to form Ba_2_Bi^3+^ *M* ^5+^O_6_. The bandgaps could be tuned to large values, because the bottom of CB would be then replaced by higher energy level d orbitals of the pentavalent cations, whereas the Bi 6s‐O 2p derived top of the VB remains the same. Indeed, Bhatia et al. reported that Ba_2_BiTaO_6_ has an ultra‐wide bandgap of 4.5 eV and K^+^ doped Ba_2_BiTaO_6_ polycrystalline pellets showed a large hole mobility of up to 30 cm^2^ V^−1^ s^−1^.^[^ ^15^ ^]^ The Bi 6s^2^‐derived VB electronic structure and tuneable bandgaps make the perovskite Ba_2_Bi*M*O_6_ (*M* = Bi, Nb, Ta) a very attractive material system as p‐type oxide semiconductors.^[^ ^14^, ^16^ ^]^ However, there is still lack of investigations of the optical and electronic properties on this material system, and even the nature and values of bandgaps remain elusive.

**Figure 1:**
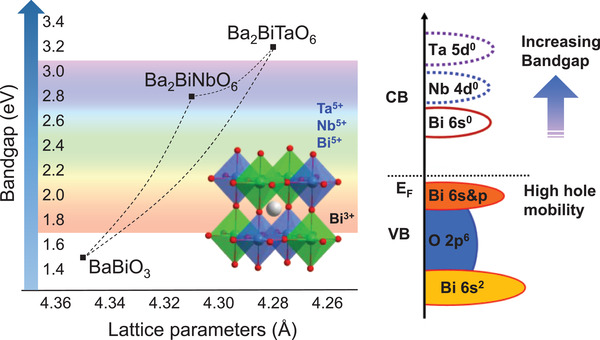
Schematic illustration of structure and bandgap modulation of perovskites Ba_2_Bi*M*O_6_ (*M* = Bi, Nb, Ta).

In this article, we present the modulation of the VB and CB structure of Ba_2_Bi*M*O_6_ (*M* = Bi, Nb, Ta) via Bi 6s^2^ state to achieve high p‐type mobility up to 21 cm^2^ V^−1^ s^−1^ and optical bandgaps varying widely from 1.5, 2.8 to 3.2 eV. A set of high‐quality Ba_2_Bi*M*O_6_ epitaxial thin films were grown by pulsed laser deposition (PLD). Hard X‐ray photoemission spectroscopy (HAXPES), X‐ray absorption spectroscopy (XAS) in conjunction with density functional theory (DFT) calculations are used to reveal insights into the electronic structures of Ba_2_Bi*M*O_6_ responsible for their appealing properties. We show that the Bi 6s^2^ state interacts strongly with O 2p to form an s–p antibonding state at the top of VB, leading to highly dispersive VB edges and thus low hole effective masses (0.4–0.7 *m*_e_) for the high hole mobility in Ba_2_Bi*M*O_6_. The large variation of the bandgaps results from the change in the energy positions of the unoccupied *s* orbital of Bi^5+^ or d orbitals of Nb/Ta that form the bottom of the CB (**Figure** **1**). Furthermore, our detailed study on the electronic structure also resolves discrepancies existing in the literature regarding the nature of the bandgaps and optical properties of Ba_2_Bi*M*O_6_. Finally, the application potential of Ba_2_Bi*M*O_6_ for optoelectronic devices were demonstrated by the fabrication of Ba_2_BiTaO_6_ – Nb doped SrTiO_3_ P–N junction diodes.

## 2 Results and Discussion

### 2.1 Thin Film Growth and Crystal Structures

**Figure 2:**
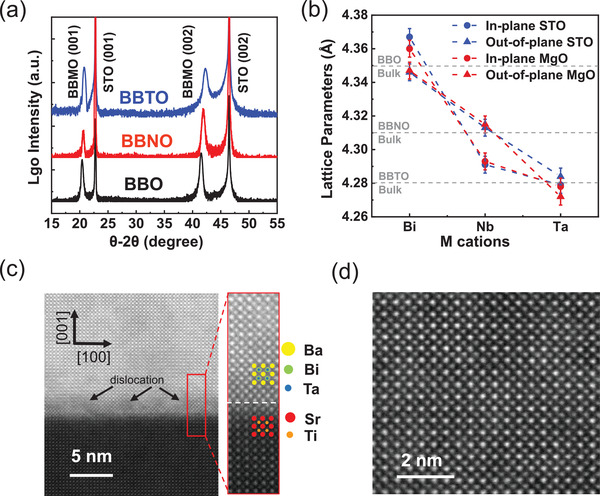
The structural and optical properties of Ba_2_Bi*M*O_6_ thin films. a) X‐ray diffraction (XRD) of Ba_2_Bi*M*O_6_ thin films grown on STO (001) substrates; b) in‐plane and out‐of‐plane lattice parameters extracted from reciprocal space mapping (RSM) of Ba_2_BiMO_6_ thin film grown on STO (001) and MgO (001) substrates; c) HAADF‐STEM image of the interface of Ba_2_BiTaO_6_ and STO, and zoom‐in into the lattice region marked by the red rectangle; d) HAADF‐STEM image of the bulk region of Ba_2_BiTaO_6_.

Ba_2_Bi*M*O_6_ (*M* = Bi, Nb, Ta) epitaxial films with thickness of ≈40 nm were grown on (001) oriented SrTiO_3_ (STO) and MgO substrates using PLD from respective targets. BaBiO_3_ (BBO) possesses a distorted perovskite structure with a pseudo‐cubic parameter of 4.35 Å. The lattice parameters slightly decrease for Ba_2_BiNbO_6_ (BBNO) (≈4.31 Å) and Ba_2_BiTaO_6_ (BBTO) (≈4.28 Å), because of the smaller ionic radii of Nb^5+^ and Ta^5+^ than that of Bi^5+^ (see schematic model in Figure 1). There is a lattice mismatch of ≈10% for Ba_2_Bi*M*O_6_ grown on STO (3.905 Å), and ≈ 2% on MgO (4.212 Å). X‐ray diffraction (XRD) *θ*–2*θ* out‐of‐plane scans in **Figure** **2**a and Figure S1 (Supporting Information) and reciprocal space mapping (RSM) in Figure S2 (Supporting Information) indicate the epitaxial growth of Ba_2_Bi*M*O_6_ thin films grown on both SrTiO_3_ and MgO substrates. Figure 2b and Table S1 (Supporting Information) summarize the in‐plane (IP) and out‐of‐plane (OP) lattice parameters of the films extracted from XRD. In general, the films grown on both substrates show the same crystalline structure and the lattice parameters decrease from Bi, Nb to Ta. The IP and OP lattice parameters are close to their respective bulk values, indicating the epitaxial strain resulting from lattice mismatch with the substrate is nearly relaxed.

Cross‐sectional scanning transmission electron microscopy (STEM) measurements were performed to further examine the interfacial structure and epitaxial relationship. A uniform film over a large lateral length scale is evident from a low‐magnification high‐angle annular dark‐field (HAADF)‐STEM image (Figure S3a, Supporting Information). Figure 2c shows an atomic‐resolution HAADF‐STEM image at the interface region of BBTO/STO viewed down to the [100] zone axis direction. Due to the large lattice mismatch, dislocations are observed in the interfacial region between BBTO and STO. However, with the increase of thin film thickness, the epitaxial strain is gradually relaxed and high crystallinity and lattice ordering are observed in the bulk region of BBTO, as shown in Figure 2d.

### 2.2 Optical and Transport Properties

**Figure 3:**
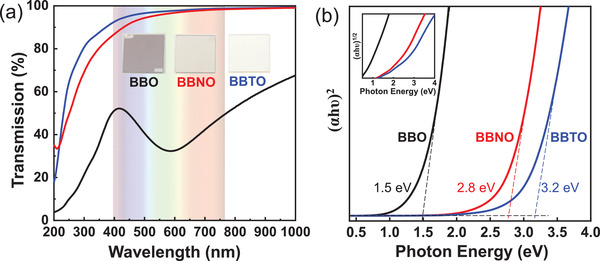
a) Optical transmittance spectra and photographs (inset) of Ba_2_Bi*M*O_6_ films grown on MgO (001) substrates; b) (*αhυ*)^2^ plot and (*αhυ*)^1/2^ plot (inset) of Ba_2_Bi*M*O_6_ films grown on MgO (001) substrates.

The optical properties of the films were measured by UV–vis–NIR absorption spectroscopy. Because of the small bandgap of STO (3.2 eV) which limits the spectrum in the UV region, we used films grown on MgO (*E*_g_ = 7.8 eV) for optical measurement. **Figure** **3**a shows the optical transmittance of 40 nm Ba_2_Bi*M*O_6_ films. BBO films show relatively low transmission of 30–40% in the visible light region. After substitution of Nb and Ta, the films show high transparency of >85% for BBNO and > 90% for BBTO from visible light towards infra‐red region. The high transparency in the visible light region makes BBNO and BBTO promising p‐type oxide semiconductors for transparent electronics.

To elucidate the bandgap values of Ba_2_Bi*M*O_6_, we further analyzed the absorption spectra using Tauc plot (Figure 3b). The extracted absorption coefficients of the films are shown in Figure S4 in the Supporting Information. Based on the slow rising of the absorption onset in the infrared light region and band structure calculations, we determined that BBO has an indirect bandgap of 0.7 eV (inset in Figure 3b showing the Tauc plot for indirect bandgap). A direct bandgap of 1.5 eV was measured. Also, a high absorption coefficient up to 3 × 10^5^ cm^–1^ in the visible light region was determined, which makes BBO an attractive p‐type perovskite material for solar energy harvesting.^[^ ^14^, ^17^ ^]^ The absorption from the infrared to visible region in BBO originates from the optical transition from the occupied antibonding state of Bi 6s‐O 2p formed at the top of VB to the unoccupied Bi 6s state at bottom of CB, which will be discussed in detail in Section 2.3. For BBNO and BBTO, the absorption band disappears and there is a gradually increased bandgap to 2.8 and 3.2 eV, which could be assigned to the substitution of unoccupied Nb 4d and Ta 5d orbitals as discussed later in Section 2.3. To further verify our concept of bandgap modulation, we also grew epitaxial films of 50% Ta^5+^ substituted BBTO (Ba_2_Bi_1.5_Ta_0.5_O_6_) and measured the optical properties (shown in Figure S5 in the Supporting Information). The direct bandgap of Ba_2_Bi_1.5_Ta_0.5_O_6_ slightly increased to 1.7 eV and the indirect bandgap increased to 1.0 eV. This demonstrates that the bandgap values could be *continuously* tuned by simply modulating the cation ratio of Bi and *M* in Ba_2_Bi*M*O_6_.

We found the undoped Ba_2_Bi*M*O_6_ in the form of both thin films and polycrystalline pellets are quite insulating, probably due to the low carrier concentration. To measure the transport properties, we used K^+^ to dope the Ba_2_Bi*M*O_6_ thin films and pellets and conducted Hall measurement at room temperature and detailed electrical properties are summarized in Table S2 in the Supporting Information. A high hole mobility of 11.3 cm^2^ V^−1^ s^−1^ was obtained for 5% K doped BBO (K_0.05_Ba_0.95_BiO_3_) thin films, and 14.1 and 21.0 cm^2^ V^−1^ s^−1^ for 20% K doped BBNO and BBTO polycrystalline pellets respectively, corroborating the success of using a Bi^3+^ 6s^2^ modulated VBM to achieve high mobility p‐type oxides. We also compare the properties of Ba_2_Bi*M*O_6_ with other p‐type oxide semiconductors in **Table** **1**. It is obvious that Ba_2_Bi*M*O_6_ outperforms most of the other p‐type oxides in terms of mobility. The high hole mobility, together with the continuously tunable bandgaps from 1.5 to 3.2 eV make Ba_2_Bi*M*O_6_ promising materials as solar energy absorber in PV and also for transparent p‐type semiconducting layers for p‐channel TFTs and P–N heterojunctions.

**Table 1:** Comparison between Ba_2_Bi*M*O_6_ with other p‐type oxide semiconductors in terms of bandgap, transparency and mobility

Materials	Bandgap / [eV]	Transparency / [T%]	Mobility / [cm^2^ V^−1^ s^−1^]	Hole effective mass / [*m*_e_]	Ref.
BaBiO_3_	1.5	40	11.3	0.399	This work
Ba_2_BiNbO_6_	2.8	85	14.1	0.713	This work
Ba_2_BiTaO_6_	3.2	90	21.0	0.590	This work
SnO	2.8	50–70	1.4–10.8	0.64	[30]
Cu_2_O	2.17	47–60	90–256	0.24	[31]
CuGaO_2_	3.6	80	0.23	2.23	[32]
SrCu_2_O_2_	3.25	60	0.46	0.79	[33]
CuBi_2_O_4_	1.8	50–85	0.02	2.29	[11a]
NiO	3.4	87	<0.05	–	[34]
BiOI	2.04	–	–	1.9	[12, 35]

### 2.3 Electronic Structure

**Figure 4:**
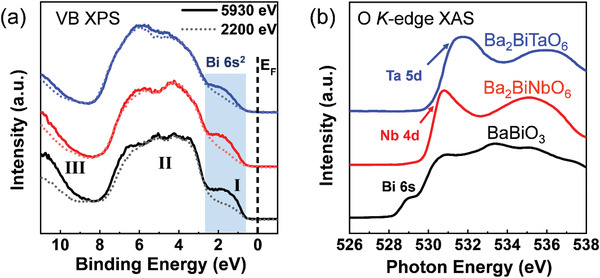
a) Valence band spectra of BaBiO_3_ (lines in black), Ba_2_BiNbO_6_ (lines in red) and Ba_2_BiTaO_6_ (lines in blue) excited with photon energy of 5930 eV (solid) and 2200 eV (dotted), three spectral regions are marked as I (0.5–2.5 eV), II (2.5–7 eV), and III (9–11 eV); b) corresponding O K‐edge XAS spectra, indicating the information about the conduction band.

A combination of HAXPES, XAS, and DFT provide crucial insights into the electronic density of states (DOS). **Figure** **4**a shows the VB spectra excited with photon energies of 2200 and 5930 eV. The VB spectra provide information about the occupied DOS weighted by the photoionization cross‐sections (PICs) of the contributing orbitals. The comparison of VB spectra excited by two photon energies allows identification of the contribution of the Bi 6s^2^ state, because the PIC of the s orbital decreases much more slowly than those of O 2p or d orbitals with increasing photon energy. As shown in Figure 4a, the three oxides show very similar VB spectra. The presence of the Bi 6s state can be clearly observed at the top of VB in the energy region of 0.5–2.5 eV (Region **I**). Spectral features in the energy region of 2.5–7 eV (Region **II**) are mainly from O 2p states. Significant contributions from Bi 6s states are also seen in Region **III** (9–11 eV) at the bottom of VB.

Figure 4b shows the O *K*‐edge XAS spectra. The O *K*‐edge XAS probes the transition from O 1s core electrons to the unoccupied states with O 2p characters hybridized with unoccupied Bi 6s/6p or d orbitals of Nb/Ta. Therefore, the O *K*‐edge XAS can be qualitatively related to the unoccupied DOS at the CB.^[^ ^18^ ^]^ Based on DFT DOS calculations, the feature centered at ≈529 eV for BBO in Figure 4b is assigned to the unoccupied Bi 6s^0^ orbital hybridized with O 2p, forming the bottom of the CB of BBO. For BBNO and BBTO, the low energy lying Bi 6s^0^ is replaced by higher energy Nb 4d^0^ and Ta 5d^0^ orbitals centered at 531 eV for BBNO and 531.7 eV for BBTO. This observation agrees well with the trend of increasing bandgaps from Bi, Nb to Ta.

**Figure 5:**
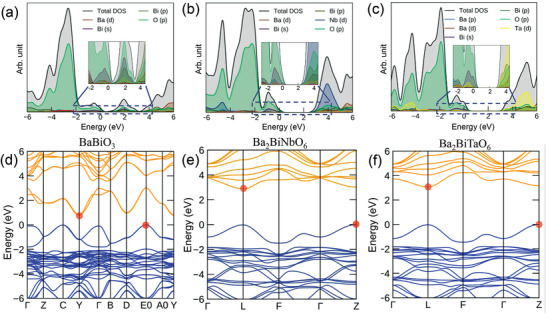
HSE06+SOC calculated a–c) DOSs and d–f) band structures for BaBiO_3_, Ba_2_BiNbO_6_, and Ba_2_BiTaO_6,_ respectively. Insets in (a)–(c) show the zoomed band edge area from −2.5 to 4.5 eV. Valence band maximum (VBM) and conduction band minimum (CBM) points are marked with red point in (d)–(f).

We further performed hybrid DFT calculations with the explicit addition of spin–orbit coupling (HSE06 + SOC) to calculate the electronic DOS near the *E*_F_. HSE06 + SOC has been proven to more accurately calculate the electronic structure of materials with strong spin–orbit coupling such as Bi based compounds.^[^ ^19^ ^]^ The calculated lattice parameters and bond lengths were minimally changed by geometry optimization (Tables S3 and S4, Supporting Information) and the calculated bandgaps match well with our experimental values (Table S5, Supporting Information). **Figure** **5**a–c plots the calculated electronic DOS for three oxides (insets showing the zoomed band‐edge region from −2.5 to 4.5 eV). The DOS with a wide energy range from −11 to 6 eV is shown in Figure S6 in the Supporting Information. Overall, the calculated DOS well reproduce the features observed in the experimental spectra of the VB photoemission and O *K*‐edge absorption. The presence of Bi 6s states with strong hybridization with O 2p at the top of VB is further confirmed by the partial DOS in the energy region from −2 to 0 eV for all three oxides. The DOS in the energy region from −6 to −2 eV (region **II** in Figure 4b) are mainly derived from O 2p with some contributions from Bi 6p or Nb/Ta d orbitals. The DOS also confirm the presence of Bi 6s states at the bottom of the VB, shown in the energy region from −11 to −9 eV (region **III** in Figure 4b). The hybridization between Bi^3+^ 6s and 6p orbitals with O 2p orbitals in Ba_2_Bi*M*O_6_ could be described via the revised lone pair (RLP) model proposed by Walsh and Payne et al.^[^ ^12^, ^20^ ^]^ the Bi^3+^ lone pair 6s^2^ states strongly hybridized with O 2p, forming Bi 6s – O 2p bonding states at the bottom of VB. The high‐energy antibonding (Bi 6s – O 2p)^*^ states further interact with unoccupied Bi 6p states due to lattice distortion, resulting in asymmetric occupied bonding states at the top of VB. The bottom of the CB of BBO is mostly derived from empty Bi 6s and 6p hybridized with O 2p. The low energy position of the empty Bi 6s results in the small indirect bandgap of 0.7 eV for BBO. On the other hand, for BBNO and BBTO, the low energy Bi 6s that formed the bottom of CB is replaced by the Nb 4d state (Figure 5b) or the Ta 5d state (Figure 5c) located at higher energy, leading to a considerable increase in the bandgap values, i.e., 2.8 eV for BBNO and 3.2 eV for BBTO. Meanwhile, the top of the VB remains nearly unchanged, because the occupied Bi 6s^2^ from Bi^3+^ is retained. The amount of increase in the bandgap is in agreement with the shift of photon energies for the features associated with Nb 4d^0^ and Ta 5d^0^ orbitals in O *K*‐edge XAS spectra.

It should be noted that different bandgap values for BBO have been reported in the literatures from indirect values of 0.3,^[^ ^21^ ^]^ 0.84 eV,^[^ ^21^ ^]^ to direct values of 1.4,^[^ ^21^ ^]^ 2.0 eV.^[^ ^14b^ ^]^ This inconsistency in the reported values of BBO bandgap might be attributed to the different computational methods or sample characteristics (e.g., polycrystalline powders or single crystalline). Our determined bandgap of 3.2 eV for BBTO is much smaller than the value of >4.5 eV reported by Bhatia et al.^[^ ^15a^ ^]^ However, considering the bandgaps of Ta_2_O_5_ (3.8 eV) and KTaO_3_ (3.6 eV) whose VB are of mainly O 2p and CB of Ta 5d states, it is reasonable to believe that the bandgap of BBTO should be smaller than 3.6 eV, because the involvement of the Bi 6s – O 2p antibonding states in BBTO would raise the VB edge to a higher energy position compared with O 2p‐dominated VB in Ta_2_O_5_ and KTaO_3_. In general, we believe that our synergistic combination of optical absorption, XPS/XAS and DFT calculations has allowed a more accurately determination of the nature and values of bandgaps for BBO and BBTO.

### 2.4 Band Structure and Effective Mass

The calculated band structures are shown in Figure 5d–f. All three Ba_2_Bi*M*O_6_ exhibit dispersive VBM in the energy region of −2 to 0 eV, providing a facile pathway for hole transport. The carrier effective mass m* is linked to mobility *(µ)* and scattering time (*τ)*, via $\mu =\frac{e\tau }{m^{*}}$. Thus, a smaller m* would result in a larger *µ*. The parabolic‐curve fitted effective mass of electrons and holes at different k‐points in the Brillion zone for BBTO, BBNO, and BBTO are summarized in **Table** **2**. As shown in Figure 5d, BBO exhibits both dispersive CBM and VBM, with a small electron effective mass (*m*_elec_*) of 0.20 *m*_e_ and hole effective mass (*m*_hole_*) of 0.40 *m*_e_. The result also indicates that BBO has an indirect bandgap of 0.78 eV and direct bandgap of 1.92 eV, in agreement with the optical absorption result (see Figure S4 in the Supporting Information). The indirect bandgap of BBO results from the optical transition between the VB at *k*‐point E0 and the CB at Y (labeled in Figure 5d). The substitution of Bi^5+^ with Nb^5+^ and Ta^5+^ not only considerably increases the bandgap values of BBNO and BBTO, but also change the bandgaps into pseudo‐direct ones (where direct and indirect gap values show small differences) because of the dramatic modification of the band structure at the CB edges. Their CB edges are less dispersive than that of BBO, due to the more localized *d* orbitals of Nb/Ta than Bi 6s. The *m*_elec_* is 0.413 *m*_e_ for BBNO and 0.406 *m*_e_ for BBTO. On the other hand, the dispersive VB edges are still maintained in BBNO and BBTO, because of the same Bi 6s^2^‐O 2p antibonding states remaining at the top of VB. The *m*_hole_* is 0.590 *m*_e_ for BBTO and 0.713 *m*_e_ for BBNO_._ The increase in *m*_hole_* compared to that of BBO is due to a decreased DOS of Bi 6s at the top of VB after the substitution of Nb and Ta, which slightly weakens the s‐orbital mediation of band dispersion. Overall, the presence of Bi 6s^2^ states successfully mediates the band dispersion at the VBM, leading to small *m*_hole_* and the observed high hole mobility.

**Table 2:** The parabolic fitted effective mass of electrons and holes at different k‐points in the Brillion zone for BaBiO_3_, Ba_2_BiNbO_6_ and Ba_2_BiTaO_6_

	BaBiO_3_		Ba_2_BiNbO_6_		Ba_2_BiTaO_6_	
*m*_hole_ ^*^/m_e_	0.40	E0	0.71	Z	0.59	Z
*m*_elec_ ^*^/m_e_	0.20	Y	0.41	L	0.41	L

### 2.5 Discussion

**Figure 6:**
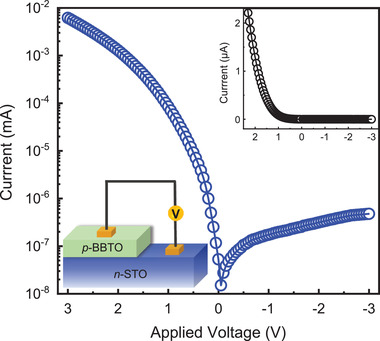
Semilogarithmic current versus voltage characteristics of the BBTO/NbSTO p–n heterojunction measured at room temperature; the inset shows the corresponding *I–V* curve in linear scale, and a schematic diagram for device measurement is also shown.

Our research reveals the origin of an observed dispersive VB and resulted high hole mobility of Ba_2_Bi*M*O_6_ perovskites by investigating their bandgap modulation and electronic structure. This would provide a viable route towards the development of high‐performance p‐type oxide semiconductors using occupied ns states from p‐block cations. As demonstrated for Ba_2_Bi*M*O_6_, the bandgap values of Bi^3+^ oxides can be modulated by choosing appropriate substitutional cations without sacrificing the dispersive 6s^2^‐modulated VB. Following this idea, other p‐block oxides with *n*s^2^ states at the VB could also be promising p‐type semiconductors with high mobility, such as the recently reported Sn^2+^ based oxides Sn_2_Nb_2_O_7_ and Sn_2_Ta_2_O_7_.^[^ ^22^ ^]^ Pb^2+^‐based oxides are also attractive choices due the similar 6s^2^‐derived VB. However, the toxicity intrinsic to Pb‐based compounds limit their further application in daily life. Additionally, the structural stability and compositional flexibility of perovskites allow us to modulate bandgap values for different applications. For example, BBO with a relatively small bandgap of 1.5 eV could be used as photocathode for solar water splitting, while the transparent BBNO and BBTO could work as p‐type channel materials for transparent TFTs or hole transport layers for photovoltaics. Bi‐excess Ba_2_Bi*_x_* Nb_2−_ *_x_* O_6_ and Ba_2_Bi*_x_* Ta_2−_ *_x_* O_6_ (1 < *x* ≤ 2) are also ideal platforms for developing high‐performance photocatalysts, arising from the joint advantages of lone pair s^2^ cations as chromophores, catalytically active metal d° cations, and adaptable bandgap values all together enabling efficient solar water splitting. This has been demonstrated in the examples of BiVO_4_ and Ba_2_Bi*_x_* Nb_2−_ *_x_* O_6_.^[^ ^16^, ^23^ ^]^ Furthermore, Ba_2_Bi*M*O_6_ could be combined with other n‐type perovskite oxide materials to design novel multifunctional devices. Several n‐type perovskite oxides have been developed, such as Nb‐doped SrTiO_3_ (NbSTO) and La‐doped BaSnO_3_ (LBSO). To give an example of this, we constructed a 60 nm thick BBTO on n‐type NbSTO p–n junction and measured its current−voltage (*I*−*V*) characteristics, as shown in **Figure** **6**. This diode shows a distinct rectifying behavior by applying both reverse and forward bias. The rectifying ratio is about 1.3 × 10^4^ at ±3 V, which is a high value compared to those of other oxide‐based p−n diodes.^[^ ^24^ ^]^ Our development of perovskite Ba_2_Bi*M*O_6_ would guide the rational design of p‐type semiconductor towards efficient carrier transport, adjustable compositions and variable bandgaps, and further promote the development of high‐performance oxide optoelectronics.

## 3 Conclusions

In this work, we present experimental and computational investigation on the bandgap modulation and electronic structure of perovskite oxides Ba_2_Bi*M*O_6_. The bandgap of Ba_2_Bi*M*O_6_ is could be gradually tuned in a wide range from 1.5, 2.8 to 3.2 eV by simply changing the *M* cations and their component proportion. Using HAXPES, the existences of Bi^3+^ 6s^2^ are experimentally observed at VBM in all three Ba_2_Bi*M*O_6_. The involvement of Bi^3+^ 6s^2^ states which help to maintain a dispersive valence band and thus give smaller hole effective masses compared with other oxides, leading to high hole mobilities up to 21 cm^2^ V^−1^ s^−1^. By combining optical absorption results, and experimental band edge structures from XPS, XAS, and DFT calculations, an accurate determination of the electronic structure and bandgap values of Ba_2_Bi*M*O_6_ is presented. The enlargement of bandgap values after the substitution of Nb and Ta at the *M* site, which is attributed to the involvement of d orbitals at the CBM, helps BBNO and BBTO to exhibit high optical transparency (>85%) from visible light to infra‐red region. Finally, the potential of Ba_2_Bi*M*O_6_ for the fabrication of all‐perovskite oxide devices is further confirmed by demonstrating a BBTO‐NbSTO p–n junction diode with high rectifying ratio up to 1.3 × 10^4^ at ±3 V. Our combined spectroscopic and computational methods, especially the use of varied photon energy for the probing of *s* states, could be further extended to the investigation of other p‐block oxides with *s* lone pair electrons. Our results add to the current understanding of the electronic structure of Bi‐based perovskite oxides and provide guidance for the development of p‐type oxide semiconductors with high mobilities.

## 4 Experimental Section

Epitaxial BaBiO_3_, Ba_2_BiNbO_6_, and Ba_2_BiTaO_6_ thin films were grown on double‐side polished (001)‐oriented SrTiO_3_ substrates and (001)‐oriented MgO substrates by PLD from respective targets. Laser ablation was performed at a repetition rate of 1 Hz and an energy density of 1.0 J cm^−2^ with a 248 nm KrF excimer laser. Films were grown at a substrate temperature of 550 °C. The oxygen partial pressure during growth was 1 Pa. The polycrystalline‐pellets of 20% K^+^ doped Ba_2_BiNbO_6_ and Ba_2_BiTaO_6_ were prepared using a solid‐state synthesis method. Briefly, stoichiometric amounts of K_2_CO_3_ ((99.98%, Alfa Aesar), BaCO_3_ (99.95%, Alfa Aesar), Bi_2_O_3_ (99.999%, Alfa Aesar), Nb_2_O_5_ (99.985%, Alfa Aesar), and Ta_2_O_5_ (99.993%, Alfa Aesar) were thoroughly ground with agate mortar and pestle. The mixture was firstly pressed into a pellet and heat treated in a furnace at 750 °C for 12 h. Then, the pellet was reground and heated again in a furnace at 950 °C for 12 h. The crystal structure and epitaxial relationship in the films was determined by high‐resolution XRD using a PANalytical four‐circle diffractometer in *θ*–2*θ* scans and reciprocal space maps (RSM) mode. Cross‐sectional STEM specimens were prepared with an FEI Helios dual‐beam focused ion beam/scanning electron microscope using a standard lift‐out approach. The FIB‐prepared samples were examined using an FEI Titan80‐300 STEM microscope with a probe spherical aberration corrector at 300 kV for high‐angle annular dark‐field (HAADF) STEM imaging. STEM‐EDS were performed on a probe aberration‐corrected JEOL JEM‐ARM200CF at 200 kV.

Optical absorption measurements were performed at room temperature using a Cary 5000 spectrophotometer in the photon energy range of 0.5–6.0 eV. Carrier mobility measurements were performed at room temperature using a Semishare Hall effect measurement system using the van der Pauw method at a magnetic field of 0.5 T. For sample preparation, the powder samples of 20% K^+^ doped Ba_2_BiNbO_6_ and Ba_2_BiTaO_6_ were firstly pressed into pellets and sintered in a furnace at 950 °C for 12 h. The as‐sintered pellets with density of ≈5 g cm^3^ was then cut into square size (≈10 × 10 mm) and thickness of ≈1 mm using diamond saw. The electrical contacts are made at the four corners of the samples, using magnetron sputtered Au electrodes for epitaxial thin films and Ag‐ink covered Ga–In eutectics (Sigma‐Aldrich) for pellet samples. The *I–V* characteristics of the BBTO‐NbSTO heterojunction diodes were measured using a Keithley 2400 multifunctional digital source meter.

Hard XPS (HAXPES) measurements using photon energies of 5930 and 2200 eV and soft XAS measurements were all performed at the I09 beamline of the Diamond Light Source, located at the Harwell Science and Innovation Campus in Didcot, UK. HAXPES spectra were energy‐resolved and measured using a VG Scienta EW4000 high‐energy electron energy analyzer with a 56° acceptance angle. The overall energy resolution for XPS measurement was about 0.25 eV. XAS measurements were performed in total electron yield (TEY) mode. The energy resolution for O K‐edge XAS was close to 0.1 eV.

All DFT calculations were performed using the Vienna ab initio Simulation Package (VASP),^[^ ^25^ ^]^ which uses the Projector Augmented Wave (PAW) method^[^ ^26^ ^]^ to model interactions between core and valence electrons. The Heyd, Scuseria, and Ernzerhof screened hybrid functional (HSE06),^[^ ^27^ ^]^ which truncates the 25% added Hartree Fock exchange used in the hybrid PBE0 functional developed by Adamo and Barone^[^ ^28^ ^]^ for the reduction of computational cost, was used for all calculations. The *k*‐point mesh density and plane wave basis set energy cut‐off were converged to within 1 and 2 meV Atom^–1^ respectively. Geometry optimization was performed for the three structures under HSE06 to a maximum interatomic force on each atom of 0.01 eV Å^–1^. Spin–orbit coupling was included for electronic structure calculations.

### Statistical Analysis

The analysis and calibration of HAXPES data were performed using DAWN Science (Version 2.14.0, Diamond Light Source) and SpecsLab Prodigy (SPECS Surface Nano Analysis GmbH). All HAXPES data were calibrated using standard Au 4f core‐level spectra. Analysis and plotting of DFT calculated electronic density of states and band structures were performed with the sumo python package.^[^ ^29^ ^]^ Effective masses were calculated by parabolic fit of the curvature at the band edges.

## Conflict of Interest

The authors declare no conflict of interest.

## Supporting information

Supporting Information

## Data Availability

The data that support the findings of this study are available from the corresponding author upon reasonable request.
