# Supplementary material for: Modulation of the Bi3+ 6s2 Lone Pair State in Perovskites for High‐Mobility p‐Type Oxide Semiconductors
Source: Adv Sci (Weinh). 2022 Jan 7;9(6):2104141. doi: 10.1002/advs.202104141 (PMC8867164; doi:10.1002/advs.202104141)
Supplement: Supplementary file 1 — Supporting Information [file ADVS-9-2104141-s001.pdf]

ADVANCED SCIENCE
Open Access

Supporting Information

for *Adv. Sci.*, DOI 10.1002/advs.202104141

Modulation of the Bi^{3+} $6s^2$ Lone Pair State in Perovskites for High-Mobility p-Type Oxide Semiconductors

Jueli Shi, Ethan A. Rubinstein, Weiwei Li, Jiaye Zhang, Ye Yang, Tien-Lin Lee, Changdong Qin, Pengfei Yan, Judith L. MacManus-Driscoll, David O. Scanlon and Kelvin H.L. Zhang**

ADVANCED SCIENCE
Open Access

Supporting Information

for *Adv. Sci.*, DOI: 10.1002/advs.202104141

Modulation of the Bi^{3+} $6s^2$ lone pair state in perovskites for high-mobility p-type oxide semiconductors

Jueli Shi, Ethan A. Rubinstein, Weiwei Li, Jiaye Zhang, Ye Yang, Tien-Lin Lee, Changdong Qin, Pengfei Yan, Judith L. MacManus-Driscoll, David O. Scanlon, Kelvin H.L. Zhang**

Supporting Information

Modulation of the Bi^{3+} $6s^2$ lone pair state in perovskites for high-mobility p-type oxide semiconductors

Jueli Shi[1], Ethan A. Rubinstein[2], Weiwei Li[3,4], Jiaye Zhang[1], Ye Yang[1], Tien-Lin Lee[5], Changdong Qin[6], Pengfei Yan[6], Judith L. MacManus-Driscoll[4], David O. Scanlon[2*], Kelvin H.L. Zhang[1*]

[1]State Key Laboratory of Physical Chemistry of Solid Surfaces, Collaborative Innovation Center of Chemistry for Energy Materials, College of Chemistry and Chemical Engineering, Xiamen University, Xiamen 361005, China

[2]Department of Chemistry and Thomas Young Centre, University College London, London WC1H 0AJ, United Kingdom

[3]MIIT Key Laboratory of Aerospace Information Materials and Physics, College of Science, Nanjing University of Aeronautics and Astronautics, Nanjing 211106, China

[4]Department of Materials Science and Metallurgy, University of Cambridge, 27 Charles Babbage Road, Cambridge, CB3 0FS, United Kingdom

[5]Diamond Light Source Ltd., Harwell Science and Innovation Campus, Didcot, OX11 0DE, United Kingdom

[6]Beijing Key Laboratory of Microstructure and Property of Solids, Faculty of Materials and Manufacturing, Beijing University of Technology, Beijing 100124, China

Email: d.scanlon@ucl.ac.uk and Kelvinzhang@xmu.edu.cn

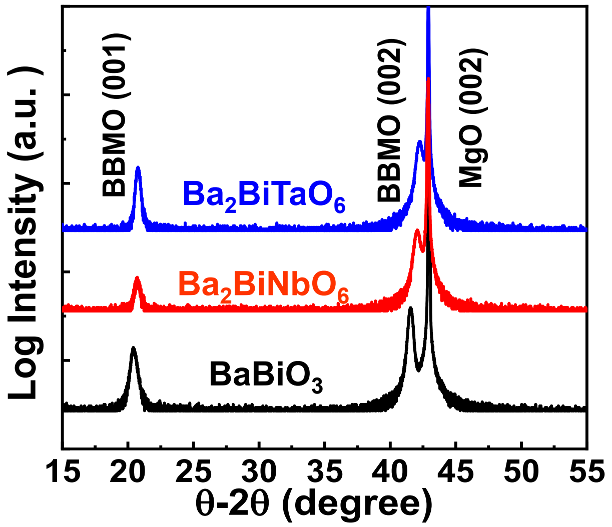

Figure S1. X-ray diffraction (XRD) of Ba_2BiMO_6 (BBMO) thin films grown on MgO (001) substrates

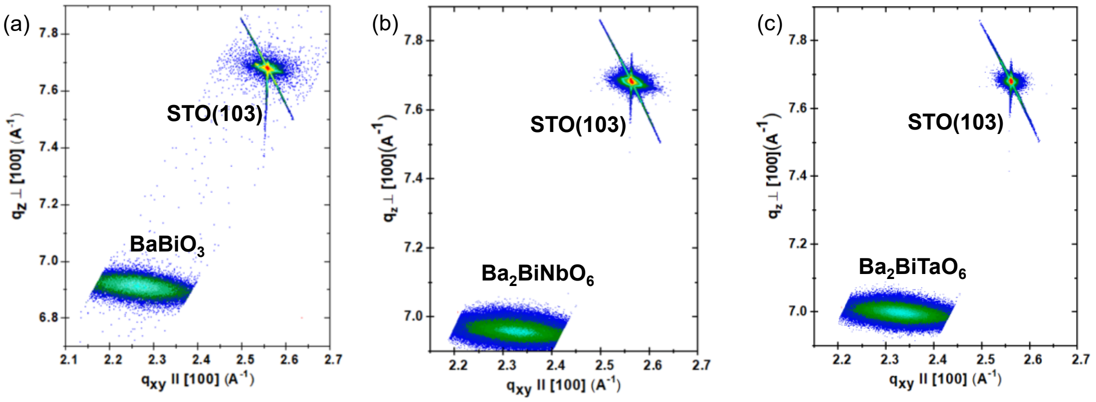

Figure S2. Reciprocal space mapping (RSM) of Ba_2BiMO_6 thin films grown on STO (001) substrate. (a) $BaBiO_3$, (b) Ba_2BiNbO_6, (c) Ba_2BiTaO_6.

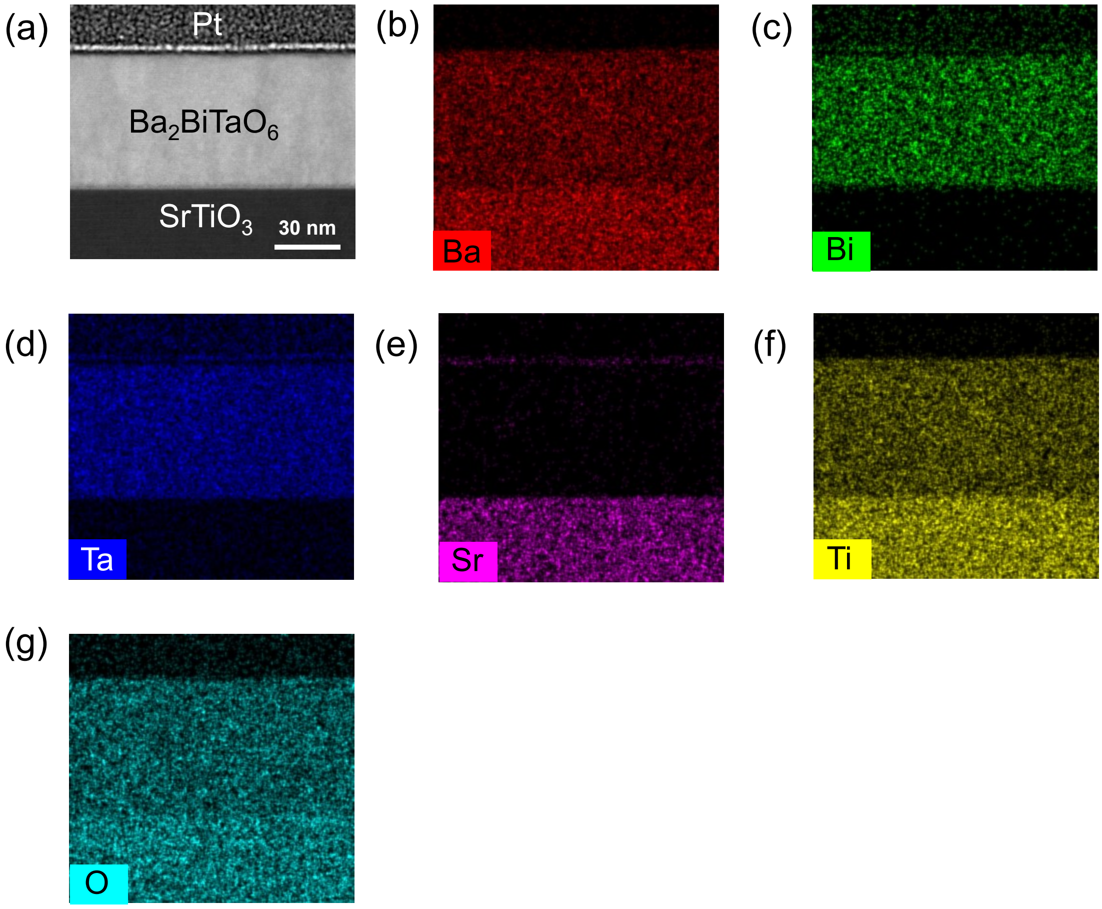

Figure S3. (a) Large-area STEM image of Ba_2BiTaO_6 film on STO(001). (b)-(g) the corresponding elemental EDS mapping of Ba, Bi, Ta, Sr, Ti, O, respectively.

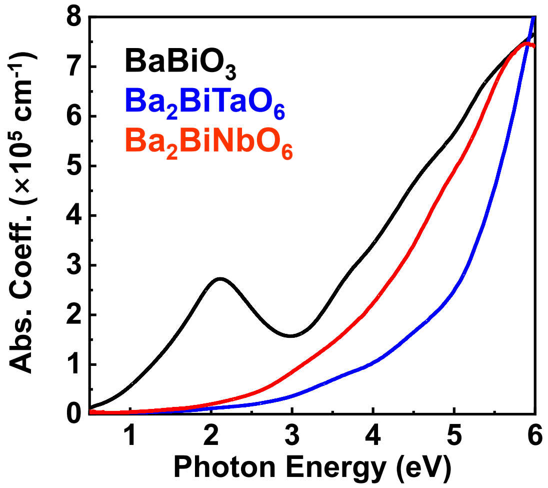

Figure S4. Optical absorption spectra of $Ba_2Bi\textit{M}O_6$ thin films grown on MgO (001) substrates.

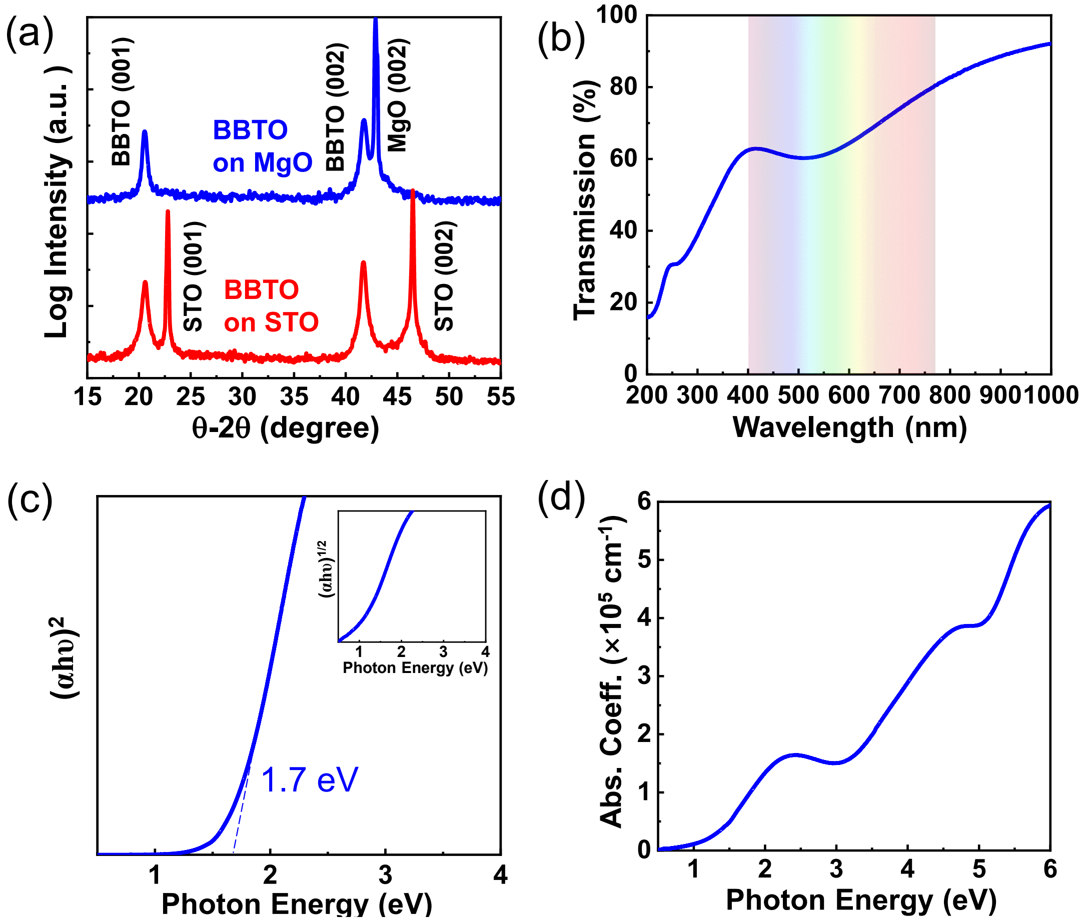

Figure S5. The crystal structure and optical properties of $Ba_2Bi_{1.5}Ta_{0.5}O_6$. (a) X-ray diffraction (XRD) of $Ba_2Bi_{1.5}Ta_{0.5}O_6$ thin films grown on MgO (001) (top, line in blue) and STO (001) (bottom, line in red) substrates; (b) optical transmittance spectra of $Ba_2Bi_{1.5}Ta_{0.5}O_6$ films grown on MgO (001) substrates; (c) $(\alpha h\upsilon)^2$ plot and $(\alpha h\upsilon)^{1/2}$ plot (inset) of $Ba_2Bi_{1.5}Ta_{0.5}O_6$ films grown on MgO (001) substrates; (d) optical absorption spectra of $Ba_2Bi_{1.5}Ta_{0.5}O_6$ thin films grown on MgO (001) substrates.

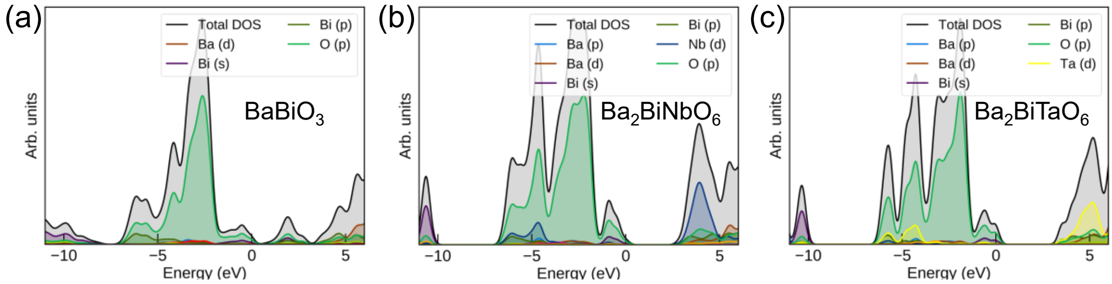

Figure S6. HSE06+SOC calculated DOS from -11 eV to 6 eV for $BaBiO_3$, Ba_2BiNbO_6 and Ba_2BiTaO_6, respectively.

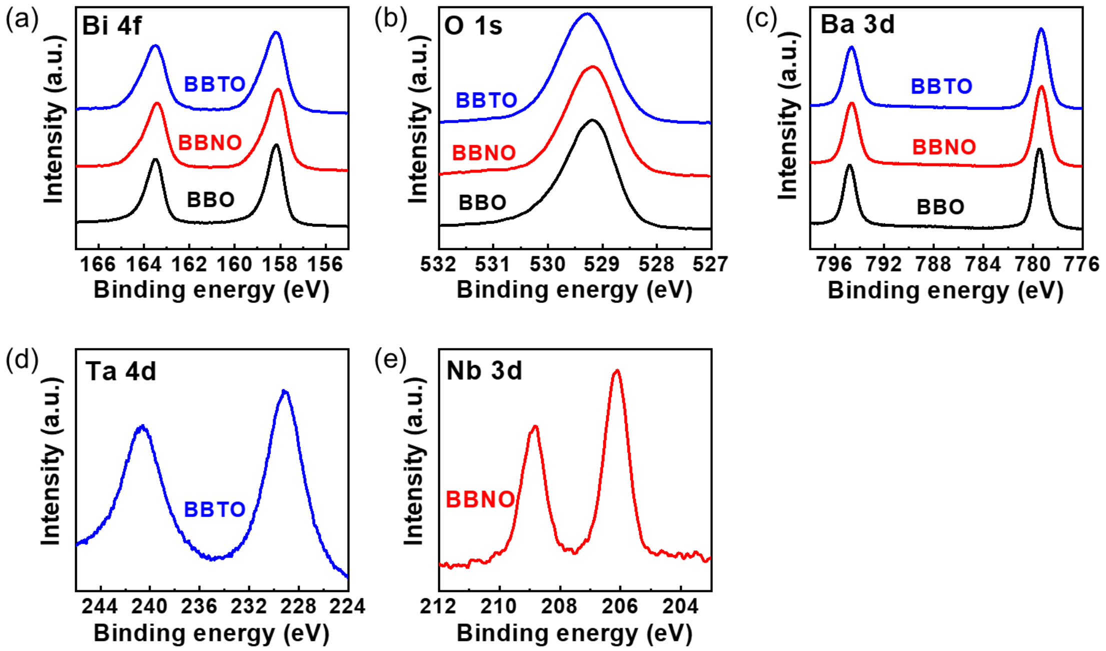

Figure S7. Core-level XPS spectra of Ba_2BiMO_6 excited with photon energy of 5930 eV. (a) Bi 4f, (b) O 1s, (c) Ba 3d, (d) Ta 4d, (e) Nb 3d.

Table S1. In-plane and out-of-plane lattice parameters of the films grown on STO (001) and MgO (001) extracted from RSMs and Bragg-Bretano scans, together with bulk values.

**Lattice parameter**	**$BaBiO_3$**	**Ba_2BiNbO_6**	**Ba_2BiTaO_6**
In-plane on STO (001) /Å	4.367	4.291	4.279
Out-of-plane on STO (001) /Å	4.346	4.313	4.284
In-plane on MgO (001) /Å	4.360	4.293	4.278
Out-of-plane on MgO (001) /Å	4.347	4.315	4.272
Bulk /Å	4.35	4.31	4.28

Table S2. Electrical Properties of for $BaBiO_3$, Ba_2BiNbO_6 and Ba_2BiTaO_6 measured at room temperature (298K) using the van der Pauw method at a magnetic field of 0.5 T.

**Electrical properties**	**5% K doped $BaBiO_3$ (thin film)**	**20% K doped Ba_2BiNbO_6 (polycrystalline pellets)**	**20% K doped Ba_2BiTaO_6 (polycrystalline pellets)**
Resistivity /Ω cm	9.64×10^0	1.58×10^3	3.68×10^3
Carrier density /cm^{-3}	5.74×10^{16}	2.82×10^{14}	8.08×10^{13}
Mobility /$cm^2\ V^{-1}\ s^{-1}$	1.13×10^1	1.41×10^1	2.10×10^1
Hall coefficient /$cm^3\ C^{-1}$	1.09×10^2	2.22×10^4	7.72×10^4
Carrier type	p-type	p-type	p-type

Table S3. Table of lattice parameters for starting and HSE06 geometry optimised structures of $BaBiO_3$ ($P2_1/c$), Ba_2BiTaO_6 (R-3R), and Ba_2BiNbO_6 (R-3R). Initial structures for $BaBiO_3$ and Ba_2BiTaO_6 obtained from the Inorganic Crystal Structure Database (ICSD).[1] * Ba_2BiNbO_6 structural model produced by substitution of Nb into Ba_2BiTaO_6 ICSD structure.

Structure	a / Å	b / Å	c / Å	α / °	*β* / °	γ / °
$BaBiO_3$ (ICSD)	6.174	6.125	10.606	90.00	125.33	90.00
$BaBiO_3$ (HSE06)	6.188 (+0.23%)	6.154 (+0.48%)	10.644 (+0.36%)	90.00	125.28 (-0.05%)	90.00
Ba_2BiTaO_6 (ICSD)	6.051	6.051	6.051	60.29	60.29	60.28
Ba_2BiTaO_6 (HSE06)	6.043 (-0.14%)	6.043 (-0.14%)	6.043 (-0.14%)	60.53 (+0.41%)	60.53 (+0.41%)	60.53 (+0.41%)
Ba_2BiNbO_6 (Model*)	6.051	6.051	6.051	60.28	60.28	60.28
Ba_2BiNbO_6 (HSE06)	6.034 (-0.28%)	6.034 (-0.28%)	6.034 (-0.28%)	60.52 (+0.40%)	60.52 (+0.40%)	60.52 (+0.40%)

Table S4. Table of bond lengths extracted from starting and HSE06 geometry optimised structures of $BaBiO_3$ ($P2_1/c$), Ba_2BiTaO_6 (R-3R), and Ba_2BiNbO_6 (R-3R). The three Bi-O bond lengths for the two distinct Bi octahedra in $BaBiO_3$ are included.

**Bond**	**Initial bond length / Å**	**HSE06 bond length / Å**	**Difference / %**
$BaBiO_3$ Bi(1)-O	2.299	2.310	0.005
$BaBiO_3$ Bi(1)-O	2.286	2.302	0.007
$BaBiO_3$ Bi(1)-O	2.289	2.306	0.007
$BaBiO_3$ Bi(2)-O	2.111	2.114	0.001
$BaBiO_3$ Bi(2)-O	2.114	2.112	-0.001
$BaBiO_3$ Bi(2)-O	2.118	2.113	-0.002
Ba_2BiTaO_6 Bi-O	2.315	2.339	0.010
Ba_2BiTaO_6 Ta-O	2.004	1.994	-0.005
Ba_2BiNbO_6 Bi-O	2.315	2.337	0.009
Ba_2BiNbO_6 Nb-O	2.004	1.988	-0.016

Table S5. Table of bandgap values determined from DFT calculation and optical measurement.

	**$BaBiO_3$**		**Ba_2BiNbO_6**		**Ba_2BiTaO_6**	
Calculational Bandgap / eV	Direct	Indirect	Direct	Indirect	Direct	Indirect
	1.92	0.78	2.90	2.88	3.10	3.08
Optical Bandgap / eV	Direct	Indirect	Direct	Indirect	Direct	Indirect
	1.5	0.7	2.8	2.6	3.2	3.0

Reference：

[1] I. Levin, National Institute of Standards and Technology, **2020**.
